# Otoneurological evaluation: definitions and scientific evidence of bedside assessment and complementary exams. Outcomes of the II Brazilian Forum of Otoneurology

**DOI:** 10.1016/j.bjorl.2025.101594

**Published:** 2025-05-13

**Authors:** Márcio Cavalcante Salmito, Ligia Oliveira Gonçalves Morganti, Alessandra Ramos Venosa, Fernando Freitas Ganança, Maurício Malavasi Ganança, Raquel Mezzalira, Roseli Saraiva Moreira Bittar, Bernardo Faria Ramos, Claudia Marques Dias, Danilo Martin Real, Denise Utsch Gonçalves, Francisco Carlos Zuma e Maia, Karen de Carvalho Lopes, Mônica Alcantara de Oliveira Santos, Paula Lobo Furtado Machado, Rafael Saba Albertino, Renato Valerio Rodrigues Cal, Ricardo Schaffeln Dorigueto, Rodrigo Cesar Silva, Rogério Castro Borges de Carvalho, Sérgio Albertino

**Affiliations:** a Universidade Federal de São Paulo (UNIFESP), Escola Paulista de Medicina, Departamento de Otorrinolaringologia e Cirurgia Cérvico-Facial, Disciplina de Otologia e Otoneurologia, São Paulo, SP, Brazil; b Universidade Federal de Minas Gerais (UFMG), Belo Horizonte, MG, Brazil; c Universidade de Brasília (UNB), Brasília, DF, Brazil; d Universidade de Campinas (UNICAMP), Disciplina de Otorrinolaringologia Cabeça e Pescoço, Campinas, SP, Brazil; e Hospital das Clínicas da Faculdade de Medicina da Universidade de São Paulo (HCFMUSP), Setor de Otoneurologia, São Paulo, SP, Brazil; f Universidade Federal do Espírito Santo (UFES), Vitória, ES, Brazil; g Hospital Madre Teresa, Belo Horizonte, MG, Brazil; h Irmandade da Santa Casa de Misericórdia de São Paulo, Faculdade de Ciências Médicas, Departamento de Otorrinolaringologia, São Paulo, SP, Brazil; i Instituto de Assistência Médica ao Servidor Público Estadual de São Paulo, São Paulo, SP, Brazil; j Clínica Equilíbrio, Brazil; k Hospital das Forças Armadas de Brasília, Brasília, DF, Brazil; l Hospital Paulista, São Paulo, SP, Brazil; m Clínica Borges de Carvalho Otorrinos, Rio de Janeiro, RJ, Brazil; n Universidade Federal Fluminense (UFF), Rio de Janeiro, RJ, Brazil; o Clínica Maia, Canoas, RS, Brazil; p Centro Universitário do Estado do Pará - CESUPA, Belém, PA, Brazil

**Keywords:** Dizziness, Otoneurology, Vestibular function tests

## Abstract

**Objective:**

To gather the technical definitions, diagnostic accuracy and scientific evidence of otoneurological exams in a succinct, easily accessible document, so that decision-making is as effective as possible.

**Method:**

This paper was the results of the II Forum of Otoneurology, held on May 27, 2018, in Sao Paulo, during the III Combined Meeting + Four Otology, as part of the Otoneurology Forums project, undertaken by the Department of Otoneurology of the Brazilian Association of Otorhinolaryngology (ABORL-CCF) since 2017. Each paper gathered here was a literature review written by specialists from all over Brazil, based on the latest practices and scientific evidence related to diagnostic tests in Otoneurology. Each author presented their paper and a debate about it took place among the participants, generating an accepted-by-all interpretation paper after each presentation. Subsequently, the papers were reviewed by representatives of the country’s Otoneurology training department for final adjustments and standardization. For each paper presented, ABORL-CCF prepared a recommendation, taking into account existing scientific evidence and the best practices, according to the experts’ opinion. Using the full paper, that was published on the website of the Brazilian Association of Otorhinolaryngology and Maxillofacial Surgery, this summarized version was written as a scientific review article.

**Outcome:**

The paper is presented as a practical, objective and complete guide on the main tests used to evaluate patients with otoneurological complaints, from bedside physical examinations to the most modern complementary tests done with sophisticated equipment.

**Conclusion:**

With this scientific review, the authors hope to aid the organization of the professional’s behavior, from the bedside assessment to the choice of assessment methods, including requisitions, accomplishments, and understanding tests and diagnostic procedures.

## Highlights

• Bedside tests are essential for evaluating vestibular function, ocular motricity and for differential diagnosis with neurological diseases.
• Complementary exams can increase the accuracy of the diagnosis and, direct the most effective treatment.
• HINTS for INFARCT is a test strongly recommended in cases of acute vertigo to differentiate, with high sensitivity, between peripheral or central origin.

## Introduction

Otoneurology is a fairly new subject, if we consider the first descriptions by Prosper Menière relating dizziness to the ear. More recently, the development of diagnosing based on scientific studies, directed physical examination and the existence of complementary exams has expanded the medical practices in this field considerably and made the knowledge and need for constant learning more robust.

To gather the scientific evidence of Otoneurology in a succinct and easily accessible document, the ABORL-CCF, through its Department of Otoneurology, developed the Otoneurology Forums project. It is aimed to provide those who treat otoneurological diseases with an organized collection of scientific evidence so that decision-making is as effective as possible.

In this II Brazilian Forum of Otoneurology, the objectives were directed to otoneurological diagnostic tests. The scientific evidence was reviewed and, together with the texts of the III Forum of Otoneurology, the current diagnostic tests were technically defined, and their diagnostic accuracy was reviewed.

## Methods

This paper was the outcome of the II Forum of Otoneurology, held on May 27, 2018, in Sao Paulo, during the III Combined Meeting + Four Otology, as part of the Otoneurology Forums project, undertaken by the Department of Otoneurology of the Brazilian Association of Otorhinolaryngology (ABORL-CCF) since 2017.

Each paper gathered here was a literature review written by specialists from all over Brazil, based on the latest practices and scientific evidence related to diagnostic tests in Otoneurology.

Each author presented their paper and a debate about it took place among the participants, generating an accepted-by-all interpretation peer-reviewed paper after each presentation. Subsequently, on September 28, 2019, the papers were fully reread and revised a second time by representatives of the country’s Otoneurology training departments for final adjustments and standardization.

The grades of evidence and levels of recommendation were defined according to the Table 1, Table 2.

**Table 1:** Strengh of action terms in guideline statements and implied levels of obligation.

Strengh	Definition	Implied Obligation
Strong recommendation	A strong recommendation means that the benefits of the recommended approach clearly the harms (or, in the case of a strong negative recommendation, that the harms clearly exceed the benefits) and that the quality of the supporting evidence is high (grade A or B).^a^ In some clearly identified circumstances, strong recommendation may be made according to lesser evidence when high-quality evidence is impossible to obtain and the antecipated benefits strongly outweight the harms.	Clinicians should follow a strong recommendation unless a clear and compelling rationale for an alterantive approach is present.
Recommendation	A recommendation means that the benefits exceed the harms (or, in the case of a negative recommendation, that the harms exceed the benefits), but the quality of evidence is not as high (grade B or C)^a^. In some clearly identified circumstances, recommendations may be made according to lesser evidence when high-quality evidence is impossible to obtain and the antecipated benefits outweight the harms.	Clinicians should also generally follow a recommendation, but should remain alert to nem information and sensitive to patient preferences.
Option	An option means either that the quality of evidence is suspect (grade D)^a^ or that well-done studies (grades A, B, or C)^a^ show little clear advantage to one approach versus another.	Clinicians should be flexible in their decision making regarding appropriate practice, although they may set bounds on alternatives; patient preference should have a substantial influencing role.

a See Table 2 for definitions of evidence grades.

**Table 2:** Aggregate grades of evidence by question type.^a^.

Grade	CEBM level	Tratment	Harm	Diagnosis	Prognosis
A	1	Systematic review^b^ of randomized trials	Systematic review^b^ of randomized trials, nested case-control studies, or observational studies with dramatic effect	Systematic review^b^ of cross-sectional studies with consistently applied reference standard and blinding	Systematic review^b^ of inception cohort studies^c^
B	2	Randomized trials or observational studies with dramatic effects or highly consistent evidence	Randomized trials or observational studies with dramatic effects or highly consistent evidence	Cross-sectional studies with consistently applied reference standard and blinding	Inception cohort studies^c^
C	3−4	Nonrandomized or historically controlled studies, including case-control and observational studies	Nonrandomized controlled cohort or follow-up study (postmarketing surveillance) with sufficient numbers to role out a common harm; case-series, case-control, or historically controlled studies	Nonconsecutive studies; case-control studies; or studies with poor, nonindependent, or inconsistently applied reference standards	Cohort study, control arm of a randomized trial, case series, or case-control studies; poor-quality prognostic cohort study
D	5	Case reports, mechanism-based reasoning, or reasoning from first principles			
X	n/a	Exceptional situations where validation studies cannot be perfomed and there is a clear preponderance of benefit over harm			

CEBM, Oxford Centre for Evidence-Based Medicine.

a Adapted from Howick and coworkers.

b A systematic review may be downgraded to level B because of study limitations, heterogeneity, or imprecision.

c A group of indiduals identified for subsequent study at an early uniform point in the course of the specified health condition or before the condition develops.

For each paper presented, ABORL-CCF prepared a recommendation, taking into account existing scientific evidence and the best practices, according to the experts’ opinion.

Using the complete paper, that was published on the website of the Brazilian Association of Otorhinolaryngology and Maxillofacial Surgery, this summarized version was written as a scientific review article.

## Outcomes

### Part 1 – bedside assessment

#### Assessment of ocular motricity^32^

##### Test of neutral gaze position (primary gaze) with and without eye fixation (detection of spontaneous nystagmus with and without gaze fixation)

**What is assessed:** Ability of the eye fixation system to keep the image of a stationary object in the fovea when the head is immobile.

Level of Evidence: A

**Level of Recommendation:** Recommended

**ABORL-CCF Recommendation:** It can be performed as a bedside assessment to diagnose diseases related to ocular motricity.

**Assessed parameters:** Ability or not to keep the eye stationary, with and without eye fixation, without pathological eye movements.^3^, ^4^

Possible outcomes:

Normal: absence of nystagmus or other eye movements.

Presence of vestibular nystagmus

Presence of other eye movements.^1^, ^2^, ^3^, ^4^

##### Eccentric gaze position test (gaze-evoked nystagmus or detection of semi-spontaneous nystagmus)

**What is assessed:** Ability of the eye fixation system for keeping the image of a stationary object in the fovea during lateral and vertical eccentric gaze.

Level of Evidence: A

**Level of Recommendation:** Recommended

**ABORL-CCF Recommendation:** It can be performed as a bedside assessment to diagnose diseases related to ocular motricity.

**Assessed parameters:** It is observed whether a nystagmus is triggered (the eye tends to return to the primary position, followed by a saccadic movement of refixation towards the target) in the eccentric gaze positions (cardinal positions) or if there is a change in the direction, shape or intensity of a previous spontaneous nystagmus.^1^, ^2^, ^3^

Possible outcomes:

Maintained eccentric fixation: normal (no nystagmus).

Presence of nystagmus (called semi-spontaneous nystagmus) or other eye movements which may be caused by different peripheral and/or central diseases.^1^, ^2^, ^3^

##### Eye movement tests

###### Optokinetic test

**What is assessed:** The visual system’s ability to reflexively induce a nystagmus by linear and continuous movement of a wide visual field.^1^, ^2^

Level of Evidence: A

**Level of Recommendation:** Recommended

**ABORL-CCF Recommendation:** It can be performed as a bedside assessment, in addition to the follow-up examination. It is useful for studying brainstem and midbrain injuries through the fast phase of nystagmus.

**Assessed parameters:** Presence and symmetry of the optokinetic reflex.^1^

Possible outcomes:

Reflexes are present and symmetrical: normal.

Asymmetry or absence of reflexes: changes in the oculomotor pathways.^1^, ^2^, ^3^, ^4^

###### Smooth pursuit test

**What is assessed:** The visual system’s ability for keeping the image of moving objects in the fovea.^1^, ^2^

Level of Evidence: A

**Level of Recommendation:** Recommended

**ABORL-CCF Recommendation:** It can be performed as a bedside assessment to diagnose diseases related to ocular motricity.

**Assessed parameters:** Ability to keep the eye fixation during object movement.^2^, ^3^, ^4^

Possible outcomes:

Normal: ability to keep the ocular fixation during object movement.

Altered: patient is not able to keep the eye fixation during object movement, suggesting changes in the oculomotor pathways.^1^, ^2^, ^3^, ^4^

###### Saccade test

**What is assessed:** The eyes’ ability to perform rapid movements in order to fix targets in the fovea that appear within the visual field.^1^, ^2^

Level of Evidence: A

**Level of Recommendation:** Recommended

**ABORL-CCF Recommendation:** It can be performed as a bedside assessment to diagnose diseases related to ocular motricity.

**Assessed parameters:** Subjective observation of speed, latency, accuracy and trajectory.

**Possible outcomes:** changes in the assessed parameters, however, without the use of objective measurement equipment, topodiagnosis is compromised.^1^, ^2^, ^3^, ^4^

###### Vergence test

**What is assessed:** The visual system’s ability to keep the image of an object simultaneously on both foveas as gaze changes from near (convergence) and far (divergence).^2^

Level of Evidence: A

**Level of Recommendation:** Recommended

**ABORL-CCF Recommendation:** It can be performed as a bedside assessment to diagnose diseases related to ocular motricity.

**Assessed parameters:** Ability or not to track the object.

Possible outcomes:

Patient can track the object: normal.

Patient cannot track the object: injury to the oculomotor pathways.^2^, ^3^, ^4^

Bedside vestibular tests

###### Bedside Head Impulse Test (bHIT)

**What is assessed**^5^, ^6^: Horizontal semicircular canals and related neural pathways through assessment of the vestibulo-ocular reflex (VOR).

**Level of Evidence:** B

**Level of Recommendation:** Strongly recommended – gold standard for bedside assessment of canalicular function.

**ABORL-CCF Recommendation:** The bedside head impulse test can be routinely performed in the assessment of patients with vestibular complaints.

Assessed parameters^6^, ^7^, ^8^:

**Keeping the gaze on the target (equivalent to the gain of the VOR):** in a healthy individual, the eye movement (VOR) triggered by the head impulse keeps the gaze fixed on the target

**Presence of corrective saccades:** individuals with ideal VOR do not manifest eye saccades when submitted to the HIT.

Possible outcomes^6^, ^7^, ^8^:

Fixed gaze at the target ahead without corrective saccades:

normal vestibular function of the lateral canals

Presence of corrective saccades due to loss of maintenance of gaze on the target:

decreased vestibular function of the lateral canals.

###### Labyrinth asymmetry tests

####### Past-pointing

**What is assessed:** Asymmetry of vestibular function. Both responses: otolithic ones, mostly, and canalicular ones, to a lesser extent, are assessed by this test.^9^, ^10^, ^11^

Level of Evidence: C

**Level of Recommendation:** Option

**ABORL-CCF Recommendation:** The physician can carry out the past-pointing test, which was one of the first tests described by Bárány in 1910, to assess the asymmetry of vestibular function. It is particularly useful in acute vestibular syndromes, when some other tests are impossible for their detection.

**Assessed parameters:** The degree of deviation of the arm and index in relation to the initial central position.^9^, ^10^, ^11^

Possible outcomes^9^, ^10^, ^11^:

Presence of point deviation: changed vestibular function. The side to which the deviation occurred indicates that it is hypofunctioning.

Absence of point deviation: normal or compensated vestibular function.

###### Head-shaking test

**What is assessed**^12^, ^13^: Screening test for vestibular asymmetry in diseases of the

peripheral and central vestibular system.

Level of Evidence: C

**Level of Recommendation:** Recommended

**ABORL-CCF Recommendation:** It is a useful test for the assessment of vestibular disorders, especially in suspected cases of unilateral vestibular hypofunction.

**Assessed parameters**^12^, ^14^, ^15^: Assessing the presence or absence of nystagmus and its characteristics.

Possible outcomes:

In the presence of nystagmus, the test is positive (changed), and in the absence of nystagmus, it is negative (normal). The changed result may indicate different conditions, depending on the characteristics of the nystagmus that is detected.

###### Vibration test

**What is assessed**^16^, ^17^: Asymmetry of labyrinth tonus in patients with unilateral vestibular hypofunctions.

Level of Evidence: C

**Level of Recommendation:** Optional

**ABORL-CCF Recommendation:** The investigation of vibration-induced nystagmus by bone stimulation can be performed with all patients with suspected asymmetries of the vestibular tonus. The result is best assessed if there is suppression of ocular fixation.

**Assessed parameters**^16^, ^17^, ^18^: Presence or not of jerk nystagmus during mastoid vibration.

Possible outcomes:

In unilateral vestibular hypofunction, there will be a horizontal nystagmus with its fast component towards the healthy side. In individuals without vestibular function asymmetry, nystagmus should not appear.

####### Hyperventilation-induced nystagmus

**What is assessed**^19^, ^20^, ^21^, ^22^: Vestibular asymmetry.

Level of Evidence: C

**Level of Recommendation:** Optional

**ABORL-CCF Recommendation:** The investigation of hyperventilation-induced nystagmus at the bedside examination is a valid option for assessing vestibular asymmetry.

**Assessed parameters:** Presence or absence of nystagmus.

**Possible outcomes**^20^, ^22^, ^23^:

Absence of nystagmus;

Presence of vestibular nystagmus with fast phase beating to the healthy side due to changes of central compensation;

Presence of nystagmus with rapid phase beating to the hypofunctioning side due to increased neural conduction in demyelinated nerve fibers or increased excitability of neural tissue.

####### Valsalva induced nystagmus

**What is assessed**^22^, ^23^: Presence of fistulas or semicircular canal dehiscence.

Level of Evidence: C

**Level of Recommendation:** Optional

**ABORL-CCF Recommendation:** The physician should conduct the test whenever there is a suspicion of some type of disease that may trigger nystagmus due to increased middle ear or intracranial pressure.

**Assessed parameters**^22^, ^23^, ^24^: If the patient has nystagmus, it means that there may be a perilymphatic fistula or a superior semicircular canal dehiscence.

**Possible outcomes**^22^, ^23^, ^24^: Horizontal nystagmus indicates some type of stimulus between the lateral semicircular canal and the middle ear, with its fast phase towards the ipsilateral side of the affected ear. Vertical and rotational nystagmus already indicate a stimulus in the superior canals, clockwise when the left ear is affected and counterclockwise when the right ear is affected, and the vertical component downwards and contralateral to the affected side.

####### Tragus compression-induced nystagmus

**What is assessed:** Presence of perilymphatic fistula.

Level of Evidence: C

**Level of Recommendation:** Optional

**ABORL-CCF Recommendation:** Assess patients with a clinical picture compatible with perilymphatic fistula.

**Assessed parameters:** The sudden pressure variation in the external auditory canal can trigger ocular deviation, nystagmus, dizziness, vertigo and body imbalance. It's a positive Hennebert’s sign.^8^, ^25^

**Possible outcomes: absence of symptoms and/or eye movements (normal).** In a positive case of fistula, it is possible to observe^8^:

**Applied positive pressure:** nystagmus towards the stimulated ear (fast component).

**Applied negative pressure:** nystagmus in the opposite direction of the stimulated ear (fast component).

###### Positional tests

**What is assessed**^1^, ^2^: The repercussions of head positioning for triggering nystagmus and vertigo.

Level of Evidence: A

**Level of Recommendation:** Recommended

**ABORL-CCF Recommendation:** It is recommended to perform the Dix-Hallpike maneuver and the roll test in patients with vestibular complaints, especially when triggered by a change in head position.

Assessed parameters^26^, ^27^:

Presence of vertigo and its characteristics.

Presence of nystagmus and its characteristics.

Possible outcomes^26^, ^27^:

Absence of nystagmus and vertigo – normal exam.

Typical BPPV nystagmus: the typical BPPV nystagmus is accompanied by vertigo, has a latency of seconds (time to onset), limited duration, increasing and decreasing intensity, exhaustion, and is fatigable to the repetition of the inducing maneuver. The direction and duration of the nystagmus indicate injury of the labyrinth, involvement of the semicircular canal (Table 3) and whether the otolith debris is adhered to the dome (cupulolithiasis) or floating in the endolymph (canalithiasis) – topographic and etiological diagnosis.

**Table 3:** Pathophysiological substrate and affected semicircular canal, according to the positional nystagmus characteristics to the Dix-Hallpike test and roll test in patients with BPPV.

Characteristics of positional nystagmus	Pathophysiological substrate and affected canal
Vertical up and torsional to the right (<1 min) to the right Dix-Hallpike	Canalithiasis of the right posterior canal
Vertical up and torsional to the left (<1 min) to the left Dix-Hallpike	Canalithiasis of the left posterior canal
Vertical down and torsional to the right (<1 min) to right and/or left Dix-Hallpike	Canalithiasis of the right anterior canal
Vertical down and torsional to the left (<1 min) to right and/or left Dix-Hallpike	Canalithiasis of the left anterior canal
More intense geotropic horizontal with right ear down to head turn maneuver	Canalithiasis of the right lateral canal
More intense geotropic horizontal with left ear down to head turn maneuver	Canalithiasis of the left lateral canal
Vertical up and torsional to the right (>1 min) to the right Dix-Hallpike	Cupulolithiasis of the right posterior canal
Vertical up and torsional to the left (>1 min) to the left Dix-Hallpike	Cupulolithiasis of the left posterior canal
Vertical down and torsional to the right (>1 min) to right and/or left Dix-Hallpike	Cupulolithiasis of the right anterior canal
Vertical down and torsional to the left (>1 min) to right and/or left Dix-Hallpike	Cupulolithiasis of the left anterior canal
More intense ageotropic horizontal with right ear down to head turn maneuver	Cupulolithiasis of the left lateral canal
More intense ageotropic horizontal with left ear down to head turn maneuver	Cupulolithiasis of the right lateral canal

Atypical nystagmus: in cases of central nervous system or neck involvement, clinical dissociation between nystagmus and vertigo, atypical or non-fatigable nystagmus upon repetition of the inducing maneuver, can usually be observed. Atypical nystagmus accompanied by typical vertigo may occur in anterior, lateral, or multicanal BPPV. – topographic and etiological diagnosis.

**Note:** If, during the diagnostic test, the patient reports only vertigo and the examiner does not observe nystagmus, we may be facing a possible BPPV (subjective BPPV).

###### Other decubitus and torsions

**What is assessed:** Tests in different decubitus and cervical torsions assess the influence of decubitus and torsions in triggering nystagmus and in the appearance or modulation of vestibular symptoms.

Level of Evidence: Exceptional situations where validating studies cannot be performed and there is a clear preponderance of benefit over harm.

**Level of Recommendation:** Recommended

**ABORL-CCF Recommendation:** Tests of different decubitus and cervical torsions are part of the group of strategies used for the characterization and differential diagnosis of vestibular disorders. Performing these tests is particularly relevant when the patient correlates the triggering of vestibular symptoms by assuming a certain position of the head, trunk or cervical torsion, as well as in the concomitance of cervical and vestibular symptoms.

**Assessed parameters**^28^, ^29^: Identification of the appearance of vestibular symptoms or nystagmus in the situations tested. In addition, the characteristics of the observed eye movements are particularly important as they contribute with information for the identification of the etiological and topographical diagnosis of the lesion.

Possible outcomes and main diagnostic hypotheses^28^, ^29^: absence of nystagmus in the different positions tested (normal); presence of nystagmus in the tested position(s), which will indicate different conditions depending on the characteristics.

***Postural tests***

***Romberg***

**What is assessed:** Static balance and sensory integration - visual, vestibular and proprioceptive.^30^

Level of Evidence: C

**Level of Recommendation:** Strongly recommended

**ABORL-CCF Recommendation:** The Romberg test is part of the physical examination and should be performed with all patients with dizziness or imbalance.

Assessed parameters:

Downward trend or oscillation may be observed;

Postural maintenance time.^1^, ^33^

Possible outcomes:

**Normal:** individual remains standing, without swaying and without falling

Changed outcomes suggest topodiagnosis of the lesion, which may involve vestibulospinal, central vestibular pathways or peripheral proprioceptive pathways reflex, as described below^1^, ^33^:

###### Unterberger-Fukuda

**What is assessed:** Dynamic balance and sensory-visual, vestibular^31^

and proprioceptive integration.

Level of Evidence: C

**Level of Recommendation:** Strongly recommended

**ABORL-CCF Recommendation:** The Unterberger-Fukuda tests should be performed in patients with dizziness or imbalance, especially in cases of acute illness.

Unterberger test:

The parameters to be assessed are^1^, ^33^, ^34^, ^35^:

presence of significant rotation.

predominant side of rotation.

Possible outcomes^1^, ^33^, ^34^, ^35^:

Healthy individuals: slight forward movement plus very slight rotation after prolonged testing (for several minutes). Such rotation occurs to the right in left-handed patients and to the left in right-handed patients.

Acute vestibular disorder: rotation in the same direction as the slow component of spontaneous nystagmus (hypofunctioning side).

Vestibular disorders in the compensation phase or chronic illness: inconclusive.

Fukuda test:

The parameters to be assessed are^1^, ^33^, ^34^, ^35^:

angle of rotation of the body relative to its vertical axis.

displacement distance.

distance of the body in relation to the starting point through the circles and lines drawn on the ground.

Possible outcomes^1^, ^33^, ^34^, ^35^:

Healthy individuals: original position. However, they can move more than 50 cm (after 50 steps) or 1 m (after 100 steps). Some normal individuals can rotate up to 30 ° (after the 50-step test) or 45 ° (after the 100-step test).

Acute vestibular hypofunction: rotation and/or displacement greater than the limits described above – in the direction of the slow phase of spontaneous nystagmus (hypofunctioning side).

Vestibular disorders in the compensation phase or chronic illness: inconclusive.

###### Cerebellar tests

####### Dysmetria detection tests

**What is assessed**^36^, ^37^: The cerebellar function in terms of automatic motricity and coordination of voluntary motricity, which are essential for proper and harmonious movement. It also assesses deep sensitivity to the exact position of each body segment and its changes in position.

Level of Evidence: A

**Level of Recommendation:** Strongly recommended

**ABORL-CCF Recommendation:** The physician should perform the finger-to-nose test for detection of dysmetria, and the reciprocating movement test for detection of diadochokinesia as part of the cerebellar function examination.

Assessed parameters:

*The assessment of dysmetria* considers the precision and range of motion. The distance or amplitude of the movement is compared to the measure considered correct for the performance of a voluntary movement.^36^, ^37^, ^38^, ^39^

*The assessment of diadochokinesia* considers the speed and rhythm of movement. The change of function is called dysdiadochokinesia or adiadochokinesia.^38^, ^39^

Possible outcomes:

*Dysmetria associated with disturbance of deep sensitivity*: patient fails to hit the target or does so imperfectly. Dysmetria clearly increases or only manifests itself when the patient closes their eyes.

*Dysmetria associated with cerebellar diseases*: the patient performs the movement exceeding the target (hypermetry) and/or in stages (movement decomposition). Dysmetria in cerebellar disease does not improve with visual control, as it does in profound sensitivity changes.

*Unilateral dysdiadochokinesia* indicates unilateral cerebellar disease, where movement is slower and poorly executed on the affected side.

*Bilateral dysdiadochokinesia* indicates diffuse cerebellar disease and progressively worsens according to the severity of the disease.^38^, ^39^

###### HINTS for INFARCT

**What is assessed**^40^: The differential diagnosis between peripheral or central vertigo in acute vestibular syndrome.

Level of Evidence: B

**Level of Recommendation:** Strongly recommended.

**ABORL-CCF Recommendation:** Test strongly recommended in cases of acute vertigo to differentiate between peripheral or central origin. The clinical changes observed in the HINTS are useful for making an early, unarmed and highly sensitive differential diagnosis in these cases.

Possible outcomes:

HINTS is an acronym derived from the naming of tests in English:

**HI** (head impulse)/**N** (gaze-evoked nystagmus)/**TS** (skew test - vertical strabismus).^40^

The peripheral syndromes (acute vestibular hypofunction) are characterized by:^40^, ^41^, ^42^

Head Impulse changed unilaterally;

More intense unidirectional horizontal nystagmus towards the fast phase side;

Skew test without gaze correction.

The central syndromes are characterized by^40^, ^41^, ^42^:

Normal Head Impulse.

Vertical nystagmus or nystagmus that changes direction in different gaze positions.

Skew test proves vertical strabismus.

INFARCT for central origin of vertigo.

**IN** (normal impulse)/**FA** (fast phase alternating)/**RCT** (refixation on cover test).

### Part 2: Otoneurological complementary exams

#### Non-vestibular Ocular Motricity Tests

##### Assessment of ocular motricity

**Indications:** Functional assessment of ocular motricity. Functional and topographic diagnosis of clinical conditions with symptoms of blurred vision, decreased visual acuity, diplopia, auditory and vestibular symptoms, imbalances and gait changes, which may indicate dysfunction of the oculomotor and/or vestibular system.

**What is assessed:** Eye movements, the neuronal structures and pathways involved when making the respective movements, and the interaction of the vestibular and oculomotor systems.^1^, ^2^

Eye movements are subdivided into^1^, ^2^:

Visual-driven ocular responses:

Optokinetic reflex

Smooth pursuit (or follow, or tracking)

Saccades

Vergencies

Visual fixation (neutral and eccentric gaze positions)**

Vestibular-driven eye movements:

Vestibulo-Ocular Reflex (VOR)

Eye movements of vestibulo-ocular interaction:

Vestibulo-ocular reflex with visual enhancement

Suppression of the vestibulo-ocular reflex

The objective recording of eye movements using oculography equipment allows obtaining more details, especially in terms of temporal and spatial resolution and oculomotor behavior, which improves the accuracy of the assessment.^43^, ^44^, ^45^, ^46^

**Level of Evidence:** B (for eye movements via video-oculography and electronystagmography)

**Level of Recommendation:** Strongly recommended

**ABORL-CCF Recommendation:** Oculomotricity assessment can be performed as part of the vestibular assessment and/or in the presence of visual or balance complaints. Assessment of visual and vestibular eye movements is usually performed as a step in the oculography examination (video or electro-oculography/nystagmography).

Assessed parameters^1^, ^2^, ^43^, ^44^, ^45^, ^46^:

##### Assessment of saccade movements

Accuracy or Precision (%) - Ability to hit the target, considering the range of motion. When the eyes miss the target, it's called dysmetria. This can be hypometry, when the eyes fall short of the target, and hypermetry (overshoot), when the eyes go beyond the target. In both situations, there are small saccades that correct the movement, repositioning the eyes on the target.

Speed (o/s) - How quickly the eyes hit the target.

Latency (ms) - Time between exposure to the visual stimulus and the saccade to bring the gaze to the target.

Assessment of smooth pursuit:

Gain - variable used to assess the effectiveness of the pursuit system. Refers to the proportion between the eye speed and target speed, where values between 0.9 and 1.0 are expected for a normal proportion.

Assessment of optokinetic nystagmus:

Symmetry - parameter used to assess optokinetic nystagmus.

The angular speed value of the resulting slow component of nystagmus is compared when the target moves to the right and to the left.

Presence of nystagmus, saccadic intrusions, ocular displacements or anomalous eye movements.

Possible outcomes^2^, ^43^, ^44^, ^45^, ^46^:

Eye movement without changes;

Eye movement with changes in one or more assessed parameters (depending on the assessed eye movement)

Saccades:

Normal latency: 200 ms between target movement and eye movement. Increased saccade latency may result from cortical brain dysfunction.

Slowing of saccades: reduced speed, which may suggest a lesion in the pons (changes upon horizontal saccades) and/or in the midbrain (vertical saccades).

Changes in the accuracy (precision) of movement, called dysmetria (hypometria, when the patient's gaze falls short of the target; and hypermetry, when the gaze goes beyond the target) and inadequate direction, which may indicate, for example, cerebellar diseases.

Smooth pursuit:

Normal gain (ratio between performed linear eye movement and expected movement) and linear morphology of the tracing.

Presence of corrective saccades to bring the eyes back to the target (which qualitatively corresponds to the so-called type III/IV tracings);

Altered gain, which may suggest impairment of central circuits, particularly brainstem and cerebellar structures.

It should be noted that the ability to perform this movement requires attention to the pursuit of the target; this ability may be reduced with age and some medications.

Optokinetic reflex:

Normal: presence of optokinetic nystagmus to both sides symmetrically.

Asymmetries between the sides, or unilateral or bilateral absence, associated with vestibular, peripheral and central dysfunctions, and lesions that affect the visual system.

Abnormalities of this reflex are best perceived with stimuli involving the entire visual field.

Vergence:

Can track target: normal.

Unable to follow: central lesion. The centers responsible for this movement are located in the midbrain. Damage to these structures results in changes with upward gaze and usually an increase in convergence tone (excessive convergence). Pons lesion and cerebellar disease can also result from changes in vergence.

#### Visually enhanced vestibulo-ocular reflex (VVOR) and vestibulo-ocular reflex suppression (VORS) tests

**Indications:** Investigation of vestibular syndromes.

**What is assessed**^47^, ^48^: vestibulo-visual interaction.

Level of Evidence: D

**Level of Recommendation:** Option

**ABORL-CCF Recommendation:** The assessment of the vestibulo-ocular reflex, with and without visual enhancement, can be performed as a complementary test for vestibular assessment, specifically for the assessment of ocular movement of vestibular origin, contributing to the assessment of the vestibulo-visual interaction.

**Assessed parameters:** Initially, the objective of the tests is to identify the presence of compensatory saccades (catch-up saccades) in the two conditions tested, when the patient's gaze remains on the fixed target (visual enhancement) and when the gaze follows the moving target (VOR suppression). The measurement of the gain of these movements is under study for the definition of the best method for this quantitative analysis.^47^, ^48^

Possible outcomes^47^, ^48^:

In the VVOR assessment, in cases of VVO hypofunction, the eyes will move off target, in the direction of head rotation, triggering a corrective saccade back on target. In CANVAS syndrome, the changed VVOR reflects a deficit in the VOR, optokinetic reflex and smooth pursuit system. In the VORS assessment, the test is positive when the eyes leave the target by the slow phase of the VOR and a corrective saccade appears. This occurs when structures involved in smooth pursuit are compromised. Peripheral vestibular injury does not compromise the smooth pursuit or VOR cancellation.

The outcomes from these tests do not depend only on the severity of the vestibular deficit and/or central structures involved, but also on the specific frequency.

#### Vestibular function tests

***Caloric test***

**Indication:** Assessment of vestibular function.

**What is assessed:** Function of the horizontal semicircular canals.

Level of Evidence: A

**What is assessed:** Strongly recommended

**ABORL-CCF Recommendation:** The test is part of the vestibular assessment and can be done routinely.

Assessed parameters^49^, ^50^, ^51^, ^52^:

Angular velocity of slow components (AVSC).

Direction of nystagmus, its changes of rhythm, amplitude and frequency.

Symmetry of responses (LP - labyrinth predominance)

Directional preponderance

Qualitative analysis of the chart

Possible outcomes^49^, ^50^, ^51^, ^52^:

For the water test, values between 7 °/s and 52 °/s are considered normal. When the post-caloric values obtained exceed these values, the response is called vestibular hyperreflexia, and when they are below these limits is vestibular hyporeflexia. For the air test, the minimum value of AVSC is 3º/s and the maximum value is 46º/s.

Normal values for LP and DP vary according to the department and are between 18% and 33% for LP and 20% and 33% for DP with water test.^4^ In the air test, the values considered normal are LP of 19% and SD of 17%.

LP indicates asymmetry in vestibular information of peripheral or central origin.

PD may be related to the presence of spontaneous nystagmus, changes in vestibular tone due to injuries to the peripheral organ, vestibular nuclei or cerebellum. Therefore, DP is not locator and expresses a disorder somewhere in the vestibular system, whether peripheral or central.

When present, changes in rhythm (dysrhythmia) and morphology (microwriting) of nystagmus, in addition to the absence of the inhibitory effect on ocular fixation, suggest involvement of the central vestibular pathways.

#### Armed head-shaking test

**Indication:** Assessment of vestibular function.

**What is assessed:** The asymmetry of the VOR triggered by the sinusoidal acceleration of the head and its sudden deceleration, which in turn triggers a nystagmus that is kept by the velocity storage process in which several CNS structures are involved.

Level of Evidence: C

**Level of Recommendation:** Recommended

**ABORL-CCF Recommendation:** The head-shaking test is a quick test that can be added to the battery of clinical tests, however, it should not be used as a single test.

**Assessed parameters:** Test positivity is defined as the presence of at least 3 beats of nystagmus after the sudden passive stop of head rotation.^13^, ^15^, ^53^

**Possible outcomes:** In cases of unilateral vestibular lesion, a typical horizontal nystagmus can be observed, with the fast phase beating towards the healthy side. This nystagmus is short in duration and gradually subsides. In some cases, there may be a second phase, when the nystagmus is reversed.

Also in central lesions, the appearance of nystagmus that do not hit the plane in which the head stimulation is performed (horizontal), and an inferior vertical or torsional nystagmus may appear (perverted).^15^, ^54^, ^55^

#### Head-shaking suppression test (head-shaking nystagmus suppression test)

**Indication:** Assessment of the central neurological function for inhibiting the vestibular nystagmus.

**What is assessed:** The effect of head tilt on nystagmus induced by the head-shaking test. It assesses the utriculo-ocular pathway, being able to differentiate the origin of central or peripheral lesions in vertigo patients.^56^

Level of Evidence: C

**Level of Recommendation:** Optional

**ABORL-CCF Recommendation:** It can be used at the bedside assessment of patients with acute vertigo to differentiate between peripheral and central vestibular syndromes.

Assessed parameters: The slow-phase velocity of induced nystagmus (AVSC) after HST and HSTS is determined by calculating the head tilt suppression index (TSI) using the following formula^56^:

Possible outcomes^56^:

Ability to suppress post-head-shaking test nystagmus (suggestive of peripheral disease).

Inability to suppress post-head-shaking test nystagmus (suggestive of central disease).

#### vHIT: HIMP

**Indication:** Assessment of vestibular function.

**What is assessed:** Function of the horizontal and vertical semicircular canals and related neural pathways by triggering the vestibulo-ocular reflex (VOR).

Level of Evidence: B

The identification of eye movement in the first 120 ms after a quick and short passive impulse of the head is only possible in the presence of a corresponding vestibular function, therefore, it is consensual that the vHIT is a test that assesses the vestibular function. The vHIT is a test of vestibular function and not an etiological diagnostic tool.

**Level of Recommendation:** Recommended – reliable method for assessing the horizontal and vertical canals.

**ABORL-CCF Recommendation:** The vHIT can be routinely performed for assessing patients with vestibular complaints.

Assessed parameters^6^, ^7^, ^57^, ^58^, ^59^, ^60^:

**Gain:** in the healthy individual, the eye movement (VOR) triggered by the head impulse keeps the gaze fixed on the target. For this, the individual performs an eye movement equal to the movement of the head, but in the opposite direction. The mathematical ratio between eye movement and head movement is called the gain. The ideal gain value is 1.0, with a tolerance margin for the normal population, variable by sex, age and equipment used.

**Presence of saccades:** healthy individuals do not usually present saccadic eye movements when submitted to HIT. The presence of corrective saccades indicates that there is vestibular hypofunction in the tested canal (the direction to which the examiner turned the patient's head).

**Symmetry**: the relationship between the lateral (right and left), anterior (right and left), and posterior (right and left) canals.

Possible outcomes and main diagnostic hypotheses^6^, ^7^, ^57^, ^58^, ^59^, ^60^:

Gain:

Normal (gaze remains fixed on the target in front): normal vestibular function of the tested canal, with an ideal value around 1.0.

Changed (target gaze deviation): changed vestibular function of the tested channel (hypo- or hyperfunction).

Corrective saccades:

Absent: situation expected for normal gains. In changed gains, the absence of gains should raise the suspicion of technical error or central change.

Present: expected situation for changed gains. The presence of corrective saccades in tests with changed gain confirms the changed function of the tested canal. The presence of saccades in tests with normal gain can mean small gaze corrections in situations of normal gains other than 1.0 or artifacts during its performance.

Symmetry:

**Symmetrical:** similar values of gain between the sides, with variable values according to the equipment.

**Asymmetric:** disproportionate values between the sides, indicating hypo- or hyperfunction on one side.

#### vHIT: SHIMP

**Indications:** Assessment of vestibular function, identification of remaining vestibular function in the unilateral and bilateral hypofunction.

**What is assessed**^1^, ^2^: Assesses the vestibular function of the patient's semicircular canals through an antisaccade on the video head impulse test and confirms the results of the vestibulo-ocular reflex (VOR) gain observed in the HIMP (Head Impulse Paradigm).

Level of Evidence: C

**Level of Recommendation:** Optional

**ABORL-CCF Recommendation:** If the physician has the video Head Impulse Test (vHIT) resource available, performing the SHIMP can help confirm the VOR gain of the lateral semicircular canal and decrease the number of artifacts that can be found in HIMP.

**Assessed parameters:** VOR gain and presence of antisaccade.

**Possible outcomes**^61^: In patients with normal vestibular function, the impulses will trigger the VOR.

In that case, at the end of the head movement, the eyes will be at approximately the same point where the test started. As the target moved with the head, the patient needs to perform an antisaccade to refix the target on the fovea. The presence of the refixation antisaccade will represent the vestibular function of the patient's horizontal semicircular canal.

In addition to analyzing the refixation antisaccade, it is also possible to assess a second parameter, which is the VOR gain itself, and is calculated in different ways, depending on the brand of equipment used by the examiner. One advantage of the SHIMP over the HIMP is the fact that the antisaccade occurs after the head movement, not interfering with the gain calculation, which can occur with the presence of “covert” saccades.

In patients with unilateral or bilateral vestibular hypofunction, in addition to the VOR gain being below normal, the antisaccades will have their amplitude reduced or even disappear (depending on the value of the VOR gain on each side).

#### Cervical VEMP (cVEMP)

**Indication:** Assessment of vestibular function.

**What is assessed**^62^, ^63^, ^64^, ^65^: Saccule and related neural pathways (tests the functional integrity of the sacculo-colic pathway: inferior vestibular nerve, vestibular nuclei, vestibulospinal tract, accessory nerve, sternocleidomastoid muscle).

Level of Evidence: X*

*There are no studies that establish the sensitivity and specificity of cVEMP to detect changes in saccular function.

**Level of Recommendation:** Strongly recommended – gold standard for assessing the saccular function.

**ABORL-CCF Recommendation:** cVEMP can be performed as part of the vestibular assessment. It has a special indication for suspected third window syndrome, involvement of the inferior branch of the vestibular nerve, sudden deafness and Ménière's disease.

Assessed parameters^63^, ^64^, ^65^:

Presence of response

Latencies:

milliseconds (P1 or P13)

milliseconds (N1 or N23)^2^, ^7^

Interamplitudes: measurements from one peak (P1) to the other (N1). Whenever possible, correction should be made according to the strength of muscle contraction.^2^

Asymmetry index: comparison of the interamplitudes on each side. Obtained through the formula: [(AM-Am) / (AM + Am)] x 100, where AM is the largest amplitude, and Am the smallest amplitude. It is the main VEMP assessment parameter and varies according to the protocol used. An asymmetry above 50% is always pathological.^2^

Response thresholds: lowest sound intensity at which it is possible to trigger a response. Typically, above 80 dBHL. It is decreased in the third window syndrome.

Possible outcomes^62^, ^63^, ^64^, ^65^:

Present, bilateral, symmetric response:

With normal interamplitudes: normal saccular function.

With bilateral interamplitude decrease: suggestive of impaired bilateral sacculo-colic reflex or insufficient contraction.

With increased interamplitudes. Suggests bilateral third window syndrome. Check response thresholds.

Asymmetry of responses (Changed asymmetry index). Suggests:

Saccular hypofunction (lesser response), for example in Ménière's Disease.

Saccular hyperresponsiveness (greater response) - third window syndrome. Check interamplitudes and response thresholds.

Absence of uni or bilateral response. Suggests:

Saccular hypofunction or involvement of the inferior branch of the vestibular nerve

Absence of bilateral response may be due to insufficient contraction or indicative of central change.

Third window syndrome: Response present with increased amplitude and lowered threshold (equal to or less than 70 dB HL).

P1N1 potential latency increase: may be indicative of central involvement, for example, multiple sclerosis.

#### Ocular VEMP (oVEMP)

**Indication:** Assessment of vestibular function.

**What is assessed**^64^, ^66^: Predominantly the utricle and its neural pathways (it tests the functional integrity of the utriculo-ocular pathway: superior vestibular nerve, vestibular nuclei, medial longitudinal fasciculus, oculomotor nuclei, extrinsic ocular muscles - inferior oblique and inferior rectus).

Level of Evidence: X*

*There are no studies that establish the sensitivity and specificity of oVEMP to detect changes in the utricular function.

**Level of Recommendation:** Strongly recommended – gold standard for assessing the utricular function.

**ABORL-CCF Recommendation:** The oVEMP should be requested as part of the vestibular assessment. It has a special indication for suspected third window syndrome, involvement of the superior branch of the vestibular nerve and Ménière's disease.

Assessed parameters^64^, ^66^, ^67^, ^68^:

**Latencies:**

milliseconds (N1 or n10 or n11)

milliseconds (P1 or p15 or p16)^2^, ^8^

**Interamplitudes:** measurements from one peak (N1) to the other (P1). Whenever possible, correction should be made according to the strength of muscle contraction.^9^

**Asymmetry index:** comparison of the interamplitudes on each side. Obtained through the formula: [(AM − Am)/(AM + Am)] × 100, where AM is the largest amplitude, and Am the smallest amplitude.^6^

It is the main VEMP assessment parameter and varies according to the protocol used. An asymmetry above 50% is always pathological.

**Response thresholds:** lowest sound intensity at which it is possible to trigger a response. Typically above 80 dBHL. It is decreased in the third window syndrome.^2^, ^4^

Possible outcomes and main diagnostic hypotheses^64^, ^66^, ^67^, ^68^:

Present, bilateral, symmetric response:

With normal interamplitudes: normal utricular function.

With increased interamplitudes: suggests bilateral third window syndrome. Check response thresholds.

With decreased interamplitudes: suggestive of impaired bilateral utriculo-ocular reflex.

Asymmetry of responses. Suggests:

Utricular hypofunction (lesser response) or

Utricular hyperresponsiveness (increased response) – third window syndrome.

Check interamplitudes and response thresholds.

Absence of uni or bilateral response. Suggests:

Utricle hypofunction or involvement of the superior branch of the vestibular nerve.

Absence of bilateral response may be indicative of central change.

Response present at a threshold equal to or less than 70 dBHL, suggestive of third window syndrome (uni or bilateral).

Latency delay of the N1P1 potential may be indicative of central impairment, such as multiple sclerosis.

### Armed positional tests

**Indication:** Diagnosis of vertigo and positional nystagmus of peripheral or central origin.

**What is assessed:** It assesses the presence of nystagmus and positional vertigo in the labyrinthine, cerebellar, brainstem, vascular and cervical diseases.

Level of Evidence: A

**Level of Recommendation:** Recommended

**ABORL-CCF Recommendation:** It is recommended to perform the diagnostic Dix-Hallpike maneuver and roll test in patients with vestibular complaints, especially when triggered by changing head position. The use of equipment such as video-oculography increases the accuracy of the diagnostic maneuver.

Assessed parameters^26^, ^27^:

Presence of vertigo and its characteristics.

Presence of nystagmus and its characteristics.

Possible outcomes^26^, ^27^:

Typical BPPV nystagmus: the typical nystagmus of BPPV is accompanied by vertigo, latency, limited duration, increasing and decreasing intensity and is fatigable by the repetition of the inducing maneuver. The direction and duration of the nystagmus indicate labyrinth injury, involvement of the semicircular canal and whether the otolith debris is adhered to the dome (cupulolithiasis) or floating in the endolymph (canalithiasis) – topographic and etiological diagnosis (Table 3).

Atypical nystagmus: in cases of central nervous system or cervical involvement, clinical dissociation between nystagmus and vertigo, atypical or non- fatigable nystagmus upon repetition of the inducing maneuver, may usually be observed. Atypical nystagmus accompanied by typical vertigo may occur in anterior, lateral or multicanal BPPV – topographic and etiological diagnosis.

**Remark:** If, during the diagnostic test, the patient reports only vertigo and the examiner does not observe nystagmus, we may be facing a possible BPPV (or subjective BPPV).

## Conclusion

The knowledge within the Otoneurology field has advanced in theory and practice over recent years, accompanied by the introduction of new and improved diagnostic tools. With this review, the authors hope to aid the organization of the professional’s performance, from the bedside assessment to the choice of assessment methods, including requisitions, performance, and understanding tests and diagnostic procedures.

## Conflicts of interest

The authors declare no conflicts of interest.
